# Interpreting drug synergy in breast cancer with deep learning using target-protein inhibition profiles

**DOI:** 10.1186/s13040-024-00359-z

**Published:** 2024-02-29

**Authors:** Thanyawee Srithanyarat, Kittisak Taoma, Thana Sutthibutpong, Marasri Ruengjitchatchawalya, Monrudee Liangruksa, Teeraphan Laomettachit

**Affiliations:** 1 Bioinformatics and Systems Biology Program, School of Bioresources and Technology, King Mongkut’s University of Technology Thonburi, Bangkok, 10150 Thailand; 2 School of Information Technology, King Mongkut’s University of Technology Thonburi, Bangkok, 10140 Thailand; 3 Department of Physics, Faculty of Science, King Mongkut’s University of Technology Thonburi, Bangkok, 10140 Thailand; 4 Theoretical and Computational Physics Group, Center of Excellence in Theoretical and Computational Science, King Mongkut’s University of Technology Thonburi, Bangkok, 10140 Thailand; 5 Biotechnology Program, School of Bioresources and Technology, King Mongkut’s University of Technology Thonburi, Bangkok, 10150 Thailand; 6 National Nanotechnology Center (NANOTEC), National Science and Technology Development Agency (NSTDA), Pathum Thani, 12120 Thailand

**Keywords:** Deep neural network, Drug combination, Small-molecule inhibitors, Synergistic effects, Targeted therapy

## Abstract

**Background:**

Breast cancer is the most common malignancy among women worldwide. Despite advances in treating breast cancer over the past decades, drug resistance and adverse effects remain challenging. Recent therapeutic progress has shifted toward using drug combinations for better treatment efficiency. However, with a growing number of potential small-molecule cancer inhibitors, in silico strategies to predict pharmacological synergy before experimental trials are required to compensate for time and cost restrictions. Many deep learning models have been previously proposed to predict the synergistic effects of drug combinations with high performance. However, these models heavily relied on a large number of drug chemical structural fingerprints as their main features, which made model interpretation a challenge.

**Results:**

This study developed a deep neural network model that predicts synergy between small-molecule pairs based on their inhibitory activities against 13 selected key proteins. The synergy prediction model achieved a Pearson correlation coefficient between model predictions and experimental data of 0.63 across five breast cancer cell lines. BT-549 and MCF-7 achieved the highest correlation of 0.67 when considering individual cell lines. Despite achieving a moderate correlation compared to previous deep learning models, our model offers a distinctive advantage in terms of interpretability. Using the inhibitory activities against key protein targets as the main features allowed a straightforward interpretation of the model since the individual features had direct biological meaning. By tracing the synergistic interactions of compounds through their target proteins, we gained insights into the patterns our model recognized as indicative of synergistic effects.

**Conclusions:**

The framework employed in the present study lays the groundwork for future advancements, especially in model interpretation. By combining deep learning techniques and target-specific models, this study shed light on potential patterns of target-protein inhibition profiles that could be exploited in breast cancer treatment.

**Supplementary Information:**

The online version contains supplementary material available at 10.1186/s13040-024-00359-z.

## Background

Breast cancer is one of the most prevalent cancers, affecting over 2 million people annually worldwide and causing the highest cancer-related deaths in women. Even though breast cancer treatment has steadily improved over the past decades, drug resistance and side effects are still the major challenges for breast cancer therapy. Cancer cells develop resistance resulting from adaptive pathway rewiring (e.g., activation of compensatory or alternative proliferation pathways) in response to treatment [1–3] or acquire resistant mutations under selective pressures of treatment [4, 5]. Another obstacle is the adverse effects of cancer drugs, which may drive patients to decline an appropriate treatment dosage, resulting in the ineffective destruction of tumor cells and the development of drug resistance. To overcome the challenges, research has shifted from single-agent targeted therapies to combination treatments that use medications with distinct but synergistic mechanisms of action to overcome resistance [6–9]. In addition, each drug can be administered at lower levels in combination therapies, circumventing high-dose toxicity, which is a limitation of single-agent treatments [10, 11].

However, the number of possible combinations for testing increases dramatically with the growing number of drugs being considered [12]. As a result, there has been extensive research into computational methods for predicting the efficacy of pharmacological combinations. For example, the AstraZeneca-Sanger Drug Combination Prediction DREAM Challenge was established to encourage innovative computational algorithms to predict drug combination effects on cancer and benchmark these approaches [13]. Participants were presented with a drug combination dataset of 11,576 tests from 910 combinations across 85 molecularly defined cancer cell lines to evaluate their computational methodologies. However, the performance of the algorithms was not satisfactory, with a weighted average Pearson correlation coefficient of 0.21 to 0.39 [13].

With deep learning, a more recent model achieved prediction performance to an average of 0.73 (Pearson correlation coefficient) [14]. The deep learning model development utilized a dataset containing 23,062 drug combinations with 38 unique drugs tested against 39 human pan-cancer cell lines. With the growing number of tested drug pairs on the DrugComb database [15], another deep learning model utilizing 286,421 drug combinations across 81 cell lines achieved a Pearson correlation coefficient of 0.79 [16]. However, these deep learning models heavily relied on a large number of drug chemical structural fingerprints as their main features, which made model interpretation a challenge.

Our study utilized a smaller set of inhibitory scores, indicating the inhibitory effects of drugs on key target proteins in cancer pathways, as the input features. This choice allowed a straightforward model interpretation because individual features are meaningful (i.e., a value between 0 and 1 of each inhibitory score indicates the likelihood of a given compound acting as an inhibitor for the respective protein target). Through a systematic exploration of the input feature space, our study uncovered synergistic patterns within the target-protein inhibition profiles, which revealed helpful information for future drug combination design and provided improved therapeutic choices for breast cancer.

## Methods

### Datasets

The current study focused on developing a drug-synergy prediction model for breast cancers. A dataset of drug combinations tested on breast cancer cell lines was obtained from the DrugComb database [15, 17]. The dataset originally contained drug combination trials across 12 breast cancer cell lines. Among the 12 breast cancer cell lines, five (BT-549, MCF-7, MDA-MB-231, MDA-MB-468, and T-47D) with the highest numbers of tested drug pairs were selected (Table 1). These cell lines represent different types of heterogeneity as classified in [18] (Table S1). Overall, the drug combinations used in this study were 24,145 pairs with 98 unique drugs.

**Table 1:** Information of datasets investigated in this study

Data	Count
Number of unique drugs	98 drugs
Total number of drug combinations	24,145 pairs
Number of drug combinations tested on MCF-7	4862 pairs
Number of drug combinations tested on T-47D	4823 pairs
Number of drug combinations tested on MDA-MB-468	4799 pairs
Number of drug combinations tested on BT-549	4806 pairs
Number of drug combinations tested on MDA-MB-231	4855 pairs

### Feature construction

We aimed to use inhibition profiles of drugs against cancer-related proteins as the main features of the model because drug target information was shown to be useful for determining drug synergy [19]. However, the target information of many cancer drugs is not available. Therefore, our first step was to develop a group of models, each of which predicted the inhibitory effects of individual drugs against a selected protein.

Initially, the protein targets of 98 drugs in our dataset were collected from the DrugBank database [20], resulting in 131 targets. However, only 13 proteins (ABL1, CSF1R, EGFR, FLT1, FLT4, KDR, KIT, MCL1, NR1I2, PDGFRB, RET, TOP2A, and TUBB1), which were the most frequent targets among the 24,145 drug pairs in the dataset (Table S2), were chosen.

Next, 13 target prediction models were individually developed to predict an inhibitory effect on the respective protein by a given compound, given its structure in the SMILES format. For each target protein, the inhibitory effect of tested compounds against the target was retrieved from the PubChem database [21]. The retrieved compounds were categorized into two classes: inhibitory and non-inhibitory. These categories were defined by PubChem, where compounds with IC50 ≤ 10 μM were classified as 'active' compounds possessing inhibitory activity, and compounds labeled as 'inactive' by PubChem were classified as non-inhibitory. Table S3 lists the number of compounds in both classes for each target protein. Next, the SMILES format of the compounds and their class (inhibitory or non-inhibitory) were used to train a graph convolutional neural network using the DeepChem library [22] with the tenfold cross-validation (CV) method. The neural network contained a graph convolutional (GC) layer that received 75 atomic features and the atomic neighborhood information from each molecule. The GC layer was followed by a dense layer and an output layer. The SoftMax function was implemented at the output layer to determine a score that represents an inhibitory effect of an input compound against the target protein.

This step finally resulted in 13 individual target prediction models (one for each of the 13 protein targets). The performance of the models was reported as the receiver operating characteristic (ROC) score.

### Drug-synergy prediction model development

**Fig. 1:**
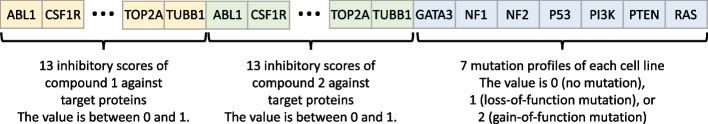
Input features of the drug-synergy prediction model. A group of graph convolutional neural network models generated 13 scores, representing inhibitory activities against 13 protein targets (the target-protein inhibition profile). To predict synergy between two drugs, the target-protein inhibition profile of each drug was concatenated, resulting in 13 × 2 = 26 features. Each feature has a value between 0 and 1. Mutation profiles of seven genes of each cell line retrieved from the Cell Model Passports and DepMap databases were also concatenated, where 0, 1, and 2 represent no mutation, loss-of-function mutation, and gain-of-function mutation, respectively

We first generated a target-protein inhibition profile for each of the 98 drugs present in the dataset to be used in developing the drug-synergy prediction model. To do this, the structure of each drug in the SMILES format was fed into each of the 13 target prediction models developed in the previous section to generate an inhibitory score against each target protein. Then, the 13 inhibitory scores of each drug (the target-protein inhibition profile) in each drug pair were combined into 26 features (13 values from each drug in the pair) (Fig. 1).

Additionally, since drug pairs have different synergistic effects when treated in other cell lines, the mutation profiles of the cell lines were also combined with the input data. The missense mutations found among the five breast cancer cell lines from Cell Model Passports [23] and DepMap [24] databases were used, and the biological function (gain-of-function or loss-of-function) was annotated with the OncoKB database [25]. This resulted in the selection of seven genes with gain-of-function or loss-of-function among the five breast cancer cell lines (Table S4). Therefore, the input data was composed of 33 features (26 inhibitory scores and seven mutation profiles) (Fig. 1). The input data and the synergy value reported in the database (the ZIP score) were used to train the drug-synergy prediction model using a 3-hidden layer neural network written with Keras.

**Fig. 2:**
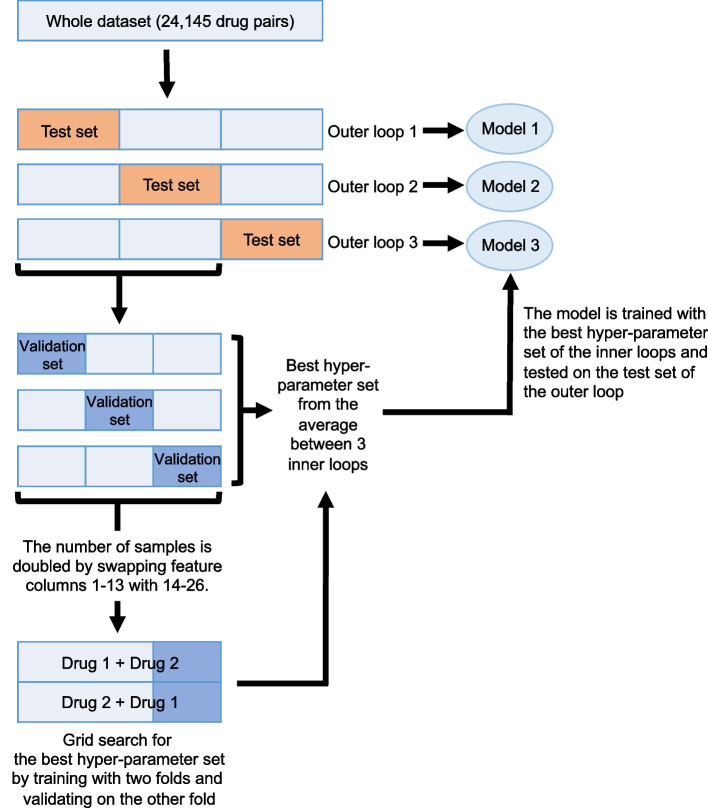
3 × 3 nested cross-validation (CV) method. 24,145 drug pairs tested on five cell lines from the DrugComb database were divided into three folds in the outer loop of the nested CV, where one fold was used as a test dataset while the other two folds were further divided into three folds in the inner loop. In each round of the inner loop, two folds were used as a training dataset, and the other fold was used as a validation set in a grid search for the best hyperparameter set. The best hyperparameter set (identified based on the average Pearson correlation coefficients obtained across each round of the three inner loops) was used to train a model, and the model was evaluated using the test set from the outer loop

In this work, the ZIP scores were normalized to have mean = 0 and variance = 1 before training. The ZIP scores greater than 0, less than 0, and equal to 0 indicate synergistic, antagonistic, and additive effects of drug pairs, respectively. A 3 × 3 nested CV method was implemented to tune the hyperparameters (inner loops) and validate the models (outer loops) (Fig. 2). The dataset (24,145 drug pairs across five breast cancer cell lines) was divided into three folds. Stratification was applied to ensure an equal representation of different cell lines within each fold. One fold was used as a test dataset in the outer loop, while the other two folds were combined and further divided into three folds in the inner loop. Two folds of the inner loop were used to tune the hyperparameters, and the other fold was a validating dataset (Fig. 2). Since drug pairs A + B and B + A should have the same synergy score, the number of drug combinations was doubled by swapping feature columns 1–13 with 14–26. We constructed the neural network model with three hidden layers. Each hidden layer had a dropout with a rate of 0.5. The output layer had a single node with 'Linear' as the activation function to predict the synergy score. We performed the grid search for the hyperparameters of the hidden layers (Table 2) using the kerashypetune package [26] to identify the best combination among all possible parameters based on the Pearson correlation coefficient between predicted ZIP scores of the validating dataset and the normalized ZIP scores from the DrugComb database. The training process was optimized by the Adam optimizer with a learning rate of 10^−5^. The best hyperparameter set indicated by the average Pearson correlation coefficients from each round of the three inner loops was used to train the model, and the model's performance was validated with the test set (outer loop).

**Table 2:** Parameters considered in hyperparameter tuning

Parameter	Possible values
The number of nodes in the first hidden layer	128, 256, 512
The number of nodes in the second hidden layer	128, 256, 512
The number of nodes in the third hidden layer	128, 256, 512
The activation function in the first hidden layer	tanh, ReLU
The activation function in the second hidden layer	tanh, ReLU
The activation function in the third hidden layer	tanh, ReLU

## Results

### Generating a target-protein inhibition profile for each drug

The selected protein targets included ABL1, CSF1R, EGFR, FLT1, FLT4, KDR, KIT, MCL1, NR1I2, PDGFRB, RET, TOP2A, and TUBB1. Most proteins are members of the receptor tyrosine kinase (RTK) family known to play a critical role in breast cancer development and metastasis [27]. The inhibitory effects of tested compounds against the 13 protein targets were used to create 13 individual target prediction models. Each model gave a score that represents the inhibitory activity of a given compound against the protein. The performance of the 13 target prediction models presented by an ROC score is shown in Table 3. The ROC scores for an independent test dataset varied from 0.6460 (the TOP2A model) to 0.9705 (the KDR model).

**Table 3:** The ROC scores of 13 target prediction models

Model	Test ROC score	Model	Test ROC score
ABL1	0.8638	MCL1	0.7567
CSF1R	0.6524	NR1I2	0.8841
EGFR	0.9110	PDGFRB	0.9227
FLT1	0.9207	RET	0.8767
FLT4	0.8997	TOP2A	0.6460
KDR	0.9705	TUBB1	0.7424
KIT	0.9363		

**Fig. 3:**
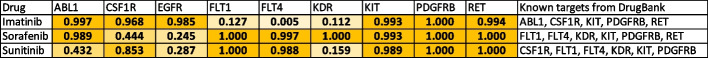
Example of inhibitory scores for three drugs (row) against some target proteins (column)

To prepare input for the drug-synergy prediction model, SMILES of 98 drugs present in the drug combination dataset were fed to each of the 13 target prediction models. Figure 3 shows the predicted scores of three example drugs. These inhibitory scores, ranging between 0 and 1, represent the likelihood of each drug inhibiting the corresponding target protein. Higher scores indicate a greater inhibitory effect on the activity of the respective protein. ABL1, CSF1R, KIT, PDGFRB, and RET have been reported to be imatinib targets in the DrugBank database. Sorefinib has been reported to antagonize FLT1, FLT4, KDR, KIT, PDGFRB, and RET. Similarly, sunitinib's targets registered in the database include CSF1R, FLT1, FLT4, KDR, KIT, and PDGFRB, which correspond with our model's predictions, except for KDR. The target-protein inhibition profiles of 98 drugs against the 13 targets are provided in Supplementary File S1.

### Developing drug-synergy prediction models

Overall, 24,145 drug pairs tested against five breast cancer cell lines obtained from the DrugComb database were used to develop the model. The inhibitory scores from the 13 target prediction models of each drug in each combination, cell lines' mutation profiles, and the reported synergy values (the normalized ZIP scores) were used to train the drug-synergy prediction model (Fig. 1 and Supplementary File S2).

**Fig. 4:**
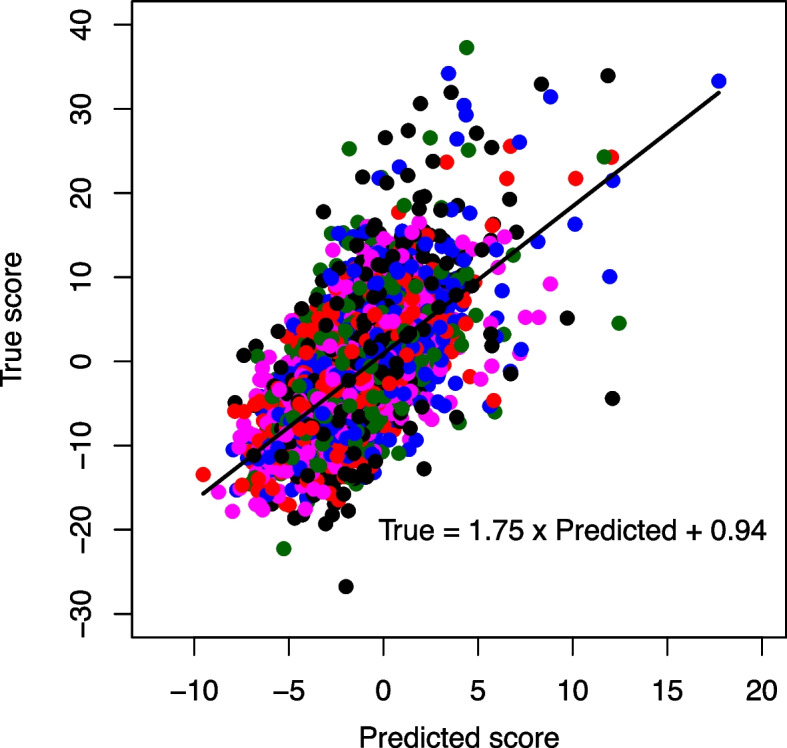
The scatter plot compares true and predicted ZIP scores from the test dataset of Model 1. The true ZIP scores were from the original values reported in the DrugComb database. BT-549 (blue); MCF-7 (red); MDA-MB-231 (magenta); MDA-MB-468 (black); T-47D (green)

A 3 × 3 nested CV method was implemented to tune the hyperparameters and evaluate the models (Fig. 2). Table 4 shows the best hyperparameter sets from the inner loops of the 3 × 3 nested CV method, which were used to train a model, and the model was tested with the test dataset from the outer loop, achieving a correlation coefficient between 0.61–0.63 and a mean squared error (MSE) ranging from 0.62–0.7 (Table 4). (Note that the synergy scores were normalized to have mean = 0 and variance = 1 before training.) Fig. 4 shows a scatter plot illustrating the relationship between experimental data and the predicted synergy scores (which were re-normalized to compare to the original data scale) from the test dataset of the model with the highest correlation coefficient of 0.63 (Model 1). When individual cell lines were considered, BT-549 and MCF-7 achieved the highest (0.67) correlation coefficient (Table 5).

**Table 4:** Hyperparameter tuning results and model performance from the 3 × 3 nested CV method

Model	The best parameters of the first hidden layer	The best parameters of the second hidden layer	The best parameters of the third hidden layer	Correlation coefficient on the test dataset	Mean squared error (MSE) on the test dataset
1	tanh; 512 nodes	ReLU; 256 nodes	ReLU; 512 nodes	0.63	0.7
2	tanh; 512 nodes	ReLU; 256 nodes	ReLU; 512 nodes	0.61	0.7
3	tanh; 512 nodes	ReLU; 512 nodes	ReLU; 128 nodes	0.62	0.62

**Table 5:** Performance of Model 1 on individual cell lines evaluated by the test dataset

Breast cancer cell line	Correlation coefficient on the test dataset	Mean squared error (MSE) on the test dataset
BT-549	0.67	0.75
MCF-7	0.67	0.59
MDA-MB-231	0.62	0.58
MDA-MB-468	0.58	0.93
T-47D	0.58	0.66

In Fig. S1, it is shown that the model produced similar predicted scores between pairs A + B and B + A (e.g., when the input feature columns 1–13 and 14–26 were swapped; see Fig. 1). The final predicted score of each drug pair used in the correlation analysis (e.g., as shown in Table 4 and 5) was derived from the average between the predicted scores of drug pairs A + B and B + A.

### Identifying synergistic patterns learned by the models

To gain further insights into the patterns underlying the observed synergistic effects of drug pairs, we systematically created target-protein inhibition profiles by assigning values of 0 or 1 to each of the 26 profile features. Particularly, drug pairs with *N* targets have values of 1 in the *N* columns and 0 in the other (26 − *N*) columns of the profile features in all possible combinations. First, we created protein inhibition profiles with the number of targets ranging from *N* = 2 to 4. The profiles were then concatenated with the cell-line mutation features. Based on the highest correlation observed in BT-549 (a triple-negative cell line) and MCF-7 (a luminal ER + cell line), we selected these two cell lines for further investigation.

**Fig. 5:**
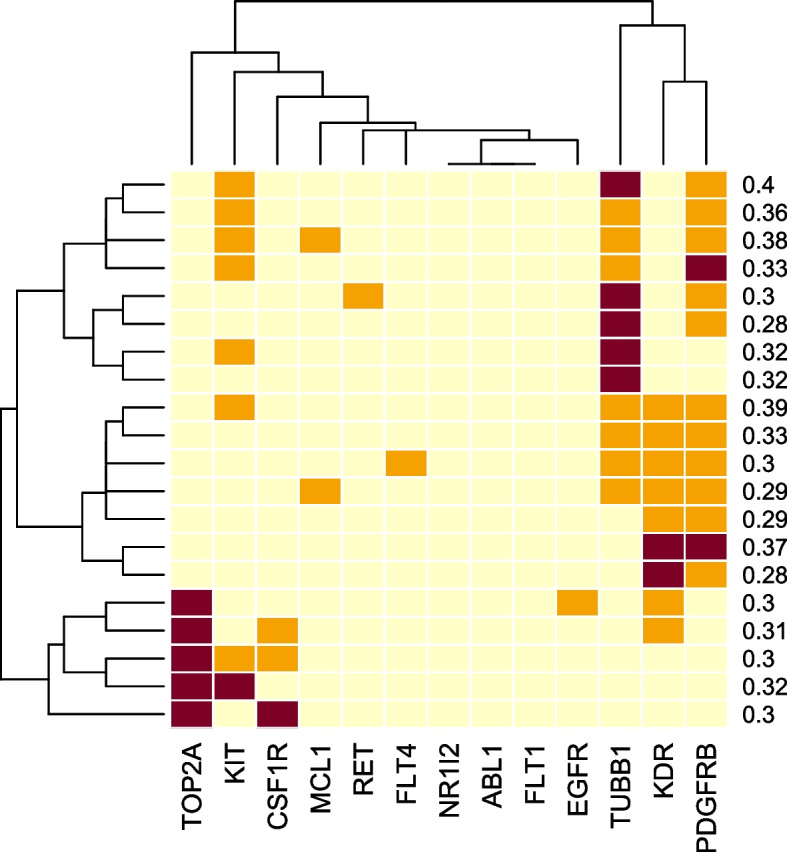
Inhibitory activities of the top 20 predicted synergy scores for BT-549 obtained from the generated inhibition profiles with 2–4 targets. The orange color indicates that the targets were inhibited with a score of 1.0 by either one of the drugs in the pair, maroon represents inhibition with a score of 1.0 by both drugs in the pair, and yellow indicates that neither of the drugs inhibited the targets (score = 0). The numbers indicate the predicted synergy scores

The generated profiles were used to predict the synergy score, which was determined by averaging the predicted scores from the three trained models. The generated profiles that yielded the top 20 predicted synergy scores were clustered into groups based on their inhibition-profile similarity using the Euclidean distance and displayed as a dendrogram. Figure 5 depicts the dendrogram of the top 20 predicted synergy scores for BT-549. The colors in the dendrogram signify the level of inhibition scores attributed to the targets. The orange color indicates that the targets were inhibited with a score of 1.0 by either one of the drugs in the pair, maroon represents inhibition with a score of 1.0 by both drugs in the pair, and yellow indicates that neither of the drugs inhibited the targets (score = 0). For example, the first row in Fig. 5 depicts a protein inhibition profile where one drug inhibited KIT, one drug inhibited PDGFRB, and both drugs inhibited TUBB1. The profile yielded the predicted synergy score of 0.4.

The dendrogram in Fig. 5 reveals two distinct groups of profiles that exhibit synergistic effects. The first group involves the inhibition among TUBB1, KDR, and PDGFRB (located in the upper right of Fig. 5). The second group comprises the inhibition among TOP2A, KIT, and CSF1R (located in the lower left of Fig. 5).

Table S5 lists drug pairs from which the models potentially captured the synergistic patterns. For instance, the combination of paclitaxel (an inhibitor of TUBB1) and sorafenib (a multi-target RTK inhibitor, including KDR and PDGFRB) yielded a ZIP score of 18.94 when tested on BT-549. Similarly, the combination of docetaxel (a TUBB1 inhibitor) and imatinib (a multi-target RTK inhibitor, including PDGFRB) exhibited a ZIP score of 14.42. Another example is the combination of dasatinib (an inhibitor of PDGFRB) and axitinib (a VEGFRs inhibitor, including KDR), achieving a ZIP score of 6.04 on BT-549. Consistent with the predictions, synthesized compounds targeting tubulin assembly, KDR, and PDGFRB displayed antiproliferative activity against a panel of cancer cell lines, including breast cancers [28–30].

The drug pairs identified as the second synergistic group by the models include, for instance, teniposide (a TOP2A inhibitor) and imatinib (a multi-target RTK inhibitor, including KIT and CSF1R), which demonstrated a ZIP score of 13.67 on BT-549 (Table S5). The potential synergy between TOP2A and RTK inhibitors has been discussed before [31]. Another combination of nilotinib (a KIT inhibitor) and sunitinib (a KIT and CSF1R inhibitor) achieved a ZIP score of 4.73. All other combinations among drug pairs whose targets included TUBB1, KDR, and PDGFRB and among drug pairs whose targets included TOP2A, KIT, and CSF1R for BT-549 are listed in Table S6-S11.

**Fig. 6:**
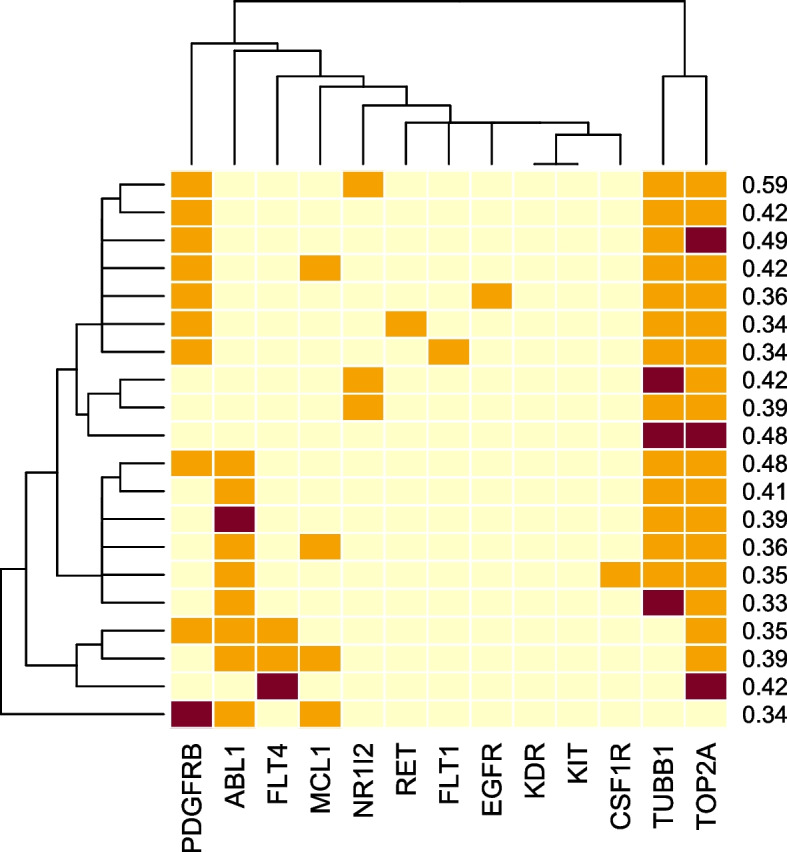
Inhibitory activities of the top 20 predicted synergy scores for MCF-7 obtained from the generated inhibition profiles with 2–4 targets. The orange color indicates that the targets were inhibited with a score of 1.0 by either one of the drugs in the pair, maroon represents inhibition with a score of 1.0 by both drugs in the pair, and yellow indicates that neither of the drugs inhibited the targets (score = 0). The numbers indicate the predicted synergy scores

For MCF-7, we observed two recognized patterns: 1) inhibition among TOP2A, TUBB1, and PDGFRB and 2) inhibition among TOP2A, TUBB1, and ABL1 (Fig. 6). Examples of drug pairs from which the model potentially captured the synergistic patterns included mitoxantrone (a TOP2A inhibitor) combined with imatinib or dasatinib (both are multi-target RTK inhibitors, including ABL1 and PDGFRB), with ZIP scores of 12.27 and 8.38, respectively, on MCF-7 (Table S12). All combinations among drug pairs whose targets included TOP2A, TUBB1, PDGFRB, and ABL1 for MCF-7 are listed in Table S13-S17.

Consistent with our predictions, synthesized multi-target compounds against tubulin polymerization and topoisomerase activity achieved antiproliferative activity against breast cancer cell lines, including MCF-7 and MDA-MB-231 [32, 33]. In addition, the combination of docetaxel (TUBB1 inhibitor) and doxorubicin (TOP2A inhibitor) has shown improved clinical outcomes for metastatic breast cancer [34, 35]. Another study showed that a multi-target compound against tubulin assembly and ABL1 significantly inhibited the growth of a panel of cancer cell lines [36].

Furthermore, we extended our analysis by creating the inhibition profiles with a higher number of targets (*N* = 4 to 6) and feeding the profiles into our models to predict the synergy scores. The predictions revealed inhibition profiles that yield high levels of synergy, as depicted in Fig. S2. For BT-549, a combination of inhibitory effects targeting TOP2A, TUBB1, PDGFRB, and EGFR was predicted to result in high synergy (Fig. S2A). It has been shown that combining a topoisomerase inhibitor with an EGFR inhibitor (e.g., gefitinib) is an effective treatment for breast and other cancer types [37–39]. Another combination involved the inhibitory activities against TOP2A, KIT, and FLT4, producing a highly synergistic effect (Fig. S2A). For MCF-7, the models predicted high synergy when combining the inhibitory effects against TOP2A, TUBB1, and PDGFRB. In one prediction, these inhibitory effects were further synergized with those against ABL1, RET, and NR1I2 (Fig. S2B).

### Investigating the effects of target-protein inhibition profiles on the synergy prediction

The framework presented in the current study relied on a two-stage prediction (a group of 13 models computed the protein-inhibition profiles for individual drugs, and then the second model utilized the profiles to predict the synergy scores between drug pairs.). Therefore, in this section, our investigation focused on the impact of the 13 protein-inhibition profiles on the performance of the synergy prediction model. We conducted a comparison of four different sets of profiles.

1. The first set was the protein-inhibition profiles generated from the 13 target prediction models (Supplementary File S1).
2. The second set was the protein-inhibition profiles generated from the 13 target prediction models created with random weights instead of trained weights. In this case, the profiles did not correspond to the correct inhibitory activities of the compounds against each target protein. Nonetheless, drugs with similar structures still possess similar profiles (Supplementary File S3).
3. The third set was the protein-inhibition profiles generated from the 13 target prediction models, which were shuffled such that each drug was associated with a profile of another random drug. Therefore, drugs with similar structures no longer possess similar profiles (Supplementary File S4).
4. The fourth set was the Morgan fingerprints. Each drug was featurized into a 2048-bit fingerprint generated from the RDKit package (Supplementary File S5).

The four sets of profiles were used to generate the input features for drug pairs. We used the model setting from Model 1 in Table 4 to compare the predictive performance on the test dataset, leave-one-cell-line-out datasets, and leave-one-drug-out datasets, as detailed in Table 6. For leave-one-cell-line-out validation, drug pairs treated on one cell line were treated as the test dataset, while drug pairs tested on the other four cell lines were used for training (predictive performances on individual leave-out cell lines are listed in Table S18). For leave-one-drug-out validation, we randomly chose 25 drugs (out of 98) for the task. For each drug left out, the drug pairs containing the leave-out drug were designated as the test set, while the remaining drug pairs were employed for training the model (predictive performances on individual leave-out drugs are listed in Table S19). The results in Table 6 demonstrate that the Morgan fingerprints were the most efficient, while the other three types of profiles exhibited similar performance, with the shuffled profiles showing the least favorable results.

**Table 6:** Comparison of predictive performance of four different sets of profiles

Profiles	Correlation coefficient			Mean squared error (MSE)		
	Test dataset	Leave-one-cell-line-out	Leave-one-drug-out	Test dataset	Leave-one-cell-line-out	Leave-one-drug-out
Protein-inhibition profiles	0.63	0.42 ± 0.07	0.42 ± 0.08	0.70	0.83 ± 0.09	1.02 ± 0.48
Protein-inhibition profiles (random weight)	0.63	0.37 ± 0.09	0.43 ± 0.08	0.68	0.87 ± 0.10	1.01 ± 0.49
Protein-inhibition profiles (shuffled)	0.56	0.43 ± 0.08	0.36 ± 0.10	0.76	0.82 ± 0.08	1.11 ± 0.54
Morgan fingerprints	0.71	0.53 ± 0.06	0.48 ± 0.12	0.56	0.72 ± 0.04	0.96 ± 0.49

The results suggest that the accuracy of the target prediction models did not have a significant impact on the synergy prediction model. The performance of the synergy prediction model that utilized the 13 target-inhibition profiles was comparable to the one that used the profiles generated from the target prediction models with random weights. This is likely because the target prediction models with random weights still generate similar profiles for drugs with similar structures. Therefore, the synergy prediction model can still learn effectively from the provided profile patterns. In contrast, the model utilizing the shuffled profiles (i.e., drug structures no longer correlated with the generated profiles) demonstrated slightly less performance in leave-one-drug-out validation. This suggests that the model struggled to generalize patterns learned from known drugs to unseen ones.

While we demonstrated that target prediction model accuracy does not hinder the learning of the synergy prediction model, it is evident that the accuracy directly influences the interpretability of learned patterns. For example, the validity of interpreting a high synergy score for drugs with inhibitory activities against TUBB1, KDR, and PDGFRB (e.g., Fig. 5) hinges on the accuracy of the 13 target prediction models. Consequently, future exploration should focus on techniques generating accurate inhibitory profiles for drugs, such as employing molecular docking to derive scores from drug interactions with target proteins.

Finally, we confirmed that structural fingerprints (e.g., Morgan fingerprints) are the most effective features in predicting synergy, as they were used successfully in previous deep learning models [14, 16]. However, unlike structural fingerprint features, our profile features offer direct interpretability. For instance, a value of 1.0 in our first feature signifies that the drug exhibits inhibitory activity against ABL1. (However, it is crucial to note that this interpretation relies on the accuracy of the ABL1 model.) Therefore, the inhibitory profiles that yield high synergy scores can directly assist in the design of novel drug combinations or guide the synthesis of multi-target compounds.

## Discussion

This study developed a deep learning model framework for predicting synergistic drug combinations specific to breast cancer cell lines and identifying potential target-protein inhibition patterns that yielded synergy among different drug combinations. Identifying protein targets of drugs is crucial to determining drug synergy [19]. However, the limited availability of protein target information hinders predicting the synergistic effects of compounds. To overcome this challenge, we addressed the issue by creating a group of target prediction models capable of generating target-protein inhibition profiles associated with 13 key proteins in breast cancer-related pathways. For this task, we chose graph neural networks (GNNs) to represent the inherent graph structure of small molecules. These networks have demonstrated superior predictive performance across various tasks [40, 41]. Training data for these models consisted of 75 atomic features and the structural topology of each compound, which were processed using the DeepChem package. These target prediction models were designed to receive compound structures in the SMILE format as input and provided 13 inhibitory scores (values between 0 and 1). These profile scores indicated the likelihood of a given compound acting as an inhibitor for the respective protein targets.

Subsequently, another deep learning model was constructed utilizing the inhibitory scores of individual compounds against 13 target proteins, along with mutation information, to predict the synergy scores for drug combinations. The model architecture consisted of five layers, including one input layer, three hidden layers, and one output layer. The input layer comprised 33 nodes. Among these, 26 nodes corresponded to the protein inhibition profiles predicted by the target prediction models for each drug pair (13 × 2 = 26), and additional seven nodes received the mutation profiles for each cell line. Dropout layers were introduced after each hidden layer to prevent overfitting. The output layer provided the predicted synergy scores, which were correlated with experimental ZIP scores obtained from DrugComb (Pearson correlation coefficients ranging between 0.61 and 0.63). When considering individual cell lines, predictions from BT-549 and MCF-7 yielded the highest correlation coefficient of 0.67.

Although our model achieved an adequate correlation, its performance was outperformed by models using structural fingerprints. For example, the model with Morgan fingerprints (correlation coefficient = 0.71) outperformed our model with the 13 target-inhibition profiles (correlation coefficient = 0.63) (Table 6). Furthermore, previously proposed deep learning models, such as DeepSynergy [14] and MatchMaker [16], achieved Pearson correlation coefficients of 0.73 and 0.69, respectively, on their datasets of pan-cancer cell lines.

However, it should be noted that a tradeoff often exists between performance and interpretability [42, 43]. The models with structural features included numerous inputs (2048 vector bits for Morgan fingerprints, 4387 chemical descriptors for DeepSynergy, and 541 chemical descriptors for MatchMaker). In addition, DeepSynergy and MatchMaker incorporated gene expression data consisting of 3984 and 972 features, respectively. Having an excessive number of features can lead to over-parameterization, making it difficult to interpret the prediction results of the models.

Unlike these models, our model relied on a smaller set of 13 × 2 = 26 features derived from predicted inhibitory activities of drugs against 13 selected key proteins, plus seven mutation features. This approach allowed a straightforward interpretation of the model since the individual features had direct biological meaning. We took advantage of this interpretability by exploring the input feature space by systematically generating target-protein inhibition profiles. As a result, we gained insights into the patterns that our models recognized as indicative of synergistic effects. Focusing on the two cell lines with the highest predictive accuracy, we found that simultaneously inhibiting TOP2A, TUBB1, PDGFRB, and EGFR resulted in high synergistic effects on BT-549. Similarly, for MCF-7, combinations involving inhibitions among TOP2A, TUBB1, PDGFRB, and ABL1 were found to generate high levels of synergy. Many predictions from the analysis are consistent with evidence from experiments showing synergistic effects. Synthesized multi-target compounds targeting tubulin assembly, KDR, and PDGFRB [28–30], combined inhibition of both tubulin polymerization and topoisomerase activity [32–35], a synthesized compound simultaneously targeting both ABL1 and tubulin assembly [36], and drug combinations targeting both EGFR and topoisomerase activity [37–39] all showed synergism in vitro or in clinical treatments. Furthermore, the model predicted additional protein-inhibition combination choices (Fig. S2), which may aid future drug combination design.

Enhancing the accuracy of the target prediction models is essential to strengthening the validity of model interpretability. This necessity arises from the inherent imbalance in the datasets used to train the target prediction models, a characteristic of bioactivity data sourced from high-throughput screening [44] (see Table S3). Addressing this problem is crucial for advancing the future development of this work. Additionally, due to the limited number of tested compounds in the database, we chose to develop a classification model for each target protein. It is interesting to investigate the possibility of using predicted IC50 values (by creating a regression model) instead of the inhibitory scores as the features in the synergy prediction model. It is also possible to prioritize other techniques that generate precise inhibitory profiles for drugs, such as utilizing molecular docking scores derived from drug interactions with target proteins.

The current model focused specifically on breast cancer, a complex disease influenced by multiple signaling pathways, which is one of the primary reasons for therapy failure. Our current model is limited to only 13 protein targets. To enhance the model's capabilities, future improvements can also involve expanding the number of protein targets associated with other key pathways, such as the PI3K/AKT/mTOR pathway, mitogen-activated protein kinases (MAPKs) pathway, and NF-κB signaling pathway. This expansion would enable the model to uncover additional underlying mechanisms related to drug synergy.

An alternative enhancement for the current study is incorporating gene expression and methylation data to provide more informative characteristics for the prediction process. In addition, exploiting more sophisticated deep learning algorithms rather than relying on a basic feedforward neural network would enhance the model's performance. One notable example is the utilization of autoencoders in AuDNNsynergy to extract representations of cancer cell line information [45]. This approach has demonstrated improved drug synergy prediction capabilities compared to earlier models. However, the future development of the models should also focus on the balance between model performance and interpretability, making it a valuable tool for selecting novel synergistic drug combinations in breast cancer treatment.

## Conclusions

The framework employed in the present study lays the groundwork for future advancements, especially in model interpretation. By tracing the synergistic interactions of compounds through their target proteins, the model framework addresses a knowledge gap in understanding the mechanism of pharmacological synergy. By combining deep learning techniques and target-specific models, this study shed light on potential patterns of target-protein inhibition profiles that could be exploited in breast cancer treatment.

### Supplementary Information

**Supplementary Material 1.**

**Supplementary Material 2.**

**Supplementary Material 3.**

**Supplementary Material 4.**

**Supplementary Material 5.**

**Supplementary Material 6.**

## Data Availability

All data generated or analyzed during this study are included in this published article and its supplementary information files and are available at https://github.com/pribnowbox/Predicting-synergy-using-protein-inhibition-profiles.
