# Supplementary material for: Interpreting drug synergy in breast cancer with deep learning using target-protein inhibition profiles
Source: BioData Min. 2024 Feb 29;17:8. doi: 10.1186/s13040-024-00359-z (PMC10905801; doi:10.1186/s13040-024-00359-z)
Supplement: Supplementary file 6 — Supplementary Material 6. [file 13040_2024_359_MOESM6_ESM.docx]

**Interpreting drug synergy in breast cancer with deep learning using target-protein inhibition profiles**

Thanyawee Srithanyarat, Kittisak Taoma, Thana Sutthibutpong, Marasri Ruengjitchatchawalya, Monrudee Liangruksa, Teeraphan Laomettachit

**
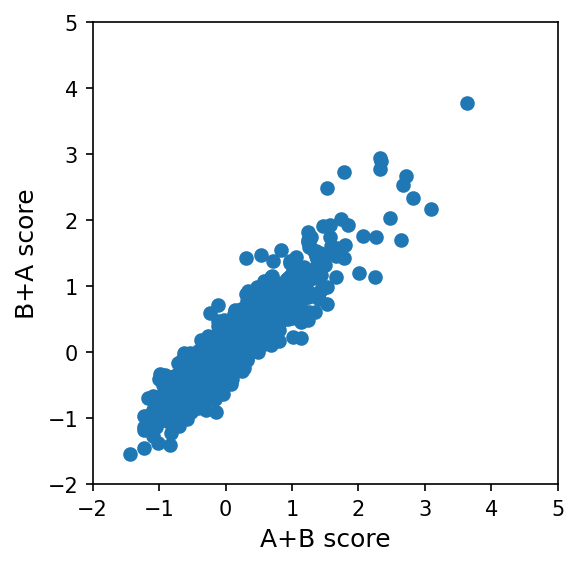
**

**Figure S1.** The comparison of predicted scores between pairs A+B and B+A from Model 1 achieved the Pearson correlation coefficient of 0.92. The final predicted score of a drug pair is the average between the predicted scores from drug pairs A+B and B+A.

**Figure S2.** Inhibitory activities of the top five predicted synergy scores for BT-549 (A) and MCF-7 (B) obtained from the generated inhibition profiles with 4-6 targets. The orange color indicates that the targets were inhibited with a score of 1.0 by either one of the drugs in the pair, maroon represents inhibition with a score of 1.0 by both drugs in the pair, and yellow indicates that neither of the drugs inhibited the targets (score = 0). The numbers indicate the predicted synergy scores.

**Table S1.** Information of cell lines investigated in this study.

| **Cell line name** | **Cell line type** | **Cell line characteristics** |
| --- | --- | --- |
| MCF-7 | Luminal A | ER^+^, PR^+/−^, HER2^−^ |
| T-47D |  |  |
| MDA-MB-468 | Basal | ER^−^, PR^−^, HER2^−^ |
| BT-549 | Claudin-low | ER^−^, PR^−^, HER2^−^ |
| MDA-MB-231 |  |  |

**Table S2.** The frequency of the top 20 protein targets present in the drug combination screening dataset. Some top proteins, such as TYMS and PTGS2, were not selected to develop into a model due to the limited number of tested compounds in PubChem.

| **Drug target** | **The number of drug pairs in the DrugComb database that target the protein (retrieved September 2020)** |
| --- | --- |
| KIT | 4167 |
| PDGFRB | 3447 |
| NR1I2 | 3369 |
| EGFR | 2623 |
| FLT4 | 2591 |
| KDR | 2591 |
| FLT1 | 2591 |
| TYMS | 2493 |
| RET | 2369 |
| ABL1 | 2338 |
| TUBB1 | 2203 |
| PTGS2 | 2128 |
| FLT3 | 1829 |
| BRAF | 1792 |
| CSF1R | 1623 |
| PDGFRA | 1623 |
| PPAT | 1619 |
| BCR | 1618 |
| TOP2A | 1574 |
| DHFR | 1512 |

**Table S3.** The number of tested compounds against each target protein from Pubchem.

| **Target** | **Inhibitory (1)** | **Non-inhibitory (0)** | **Target** | **Inhibitory (1)** | **Non-inhibitory (0)** |
| --- | --- | --- | --- | --- | --- |
| ABL1 | 1984 | 1016 | MCL1 | 1273 | 1727 |
| CSF1R | 500 | 500 | NR1I2 | 1221 | 229 |
| EGFR | 2759 | 241 | PDGFRB | 2276 | 224 |
| FLT1 | 1512 | 488 | RET | 1164 | 336 |
| FLT4 | 1100 | 400 | TOP2A | 955 | 405 |
| KDR | 3299 | 701 | TUBB1 | 920 | 80 |
| KIT | 2927 | 573 |  |  |  |

**Table S4.** Mutation profiles of seven genes among five breast cancer cell lines.

|  | **GATA3** | **NF1** | **NF2** | **P53** | **PI3K** | **PTEN** | **RAS** |
| --- | --- | --- | --- | --- | --- | --- | --- |
| BT-549 | 0 | 0 | 0 | 1 | 0 | 1 | 0 |
| MCF-7 | 1 | 0 | 0 | 0 | 2 | 0 | 0 |
| MDA-MB-231 | 0 | 1 | 1 | 1 | 0 | 0 | 2 |
| MDA-MB-468 | 0 | 0 | 0 | 1 | 0 | 0 | 0 |
| T-47D | 0 | 0 | 0 | 1 | 2 | 0 | 0 |

0, 1, and 2 represent no mutation, loss-of-function mutation, and gain-of-function mutation, respectively.

**Table S5.** Examples of drug pairs from which the models potentially captured the synergistic patterns for BT-549.

| Drug 1 | Drug 2 | Drug 1's target(s) retrieved from DrugBank | Drug 2's target(s) retrieved from DrugBank | ZIP retrieved from DrugComb |
| --- | --- | --- | --- | --- |
| PACLITAXEL | SORAFENIB | TUBB1, NR1I2 | FLT4, KDR, PDGFRB, KIT, RET, FLT1 | 18.94 |
| DOCETAXEL | SORAFENIB | TUBB1, NR1I2 | FLT4, KDR, PDGFRB, KIT, RET, FLT1 | 15.81 |
| DOCETAXEL | IMATINIB | TUBB1, NR1I2 | KIT, RET, CSF1R, ABL1, PDGFRB | 14.42 |
| DASATINIB | AXITINIB | ABL1, KIT, PDGFRB | FLT1, KDR, FLT4 | 6.04 |
| DASATINIB | PAZOPANIB HYDROCHLORIDE | ABL1, KIT, PDGFRB | FLT1, KDR, FLT4, PDGFRB, KIT | 5.49 |
| IMATINIB | SUNITINIB | KIT, RET, CSF1R, ABL1, PDGFRB | PDGFRB, FLT1, KIT, KDR, FLT4, CSF1R | 4.24 |
| TENIPOSIDE | IMATINIB | TOP2A | KIT, RET, CSF1R, ABL1, PDGFRB | 13.67 |
| DAUNORUBICIN HYDROCHLORIDE | PAZOPANIB HYDROCHLORIDE | TOP2A | FLT1, KDR, FLT4, PDGFRB, KIT | 13.04 |
| MITOXANTRONE | IMATINIB | TOP2A | KIT, RET, CSF1R, ABL1, PDGFRB | 12.47 |
| NILOTINIB | SUNITINIB | ABL1, KIT | PDGFRB, FLT1, KIT, KDR, FLT4, CSF1R | 4.73 |
| IMATINIB | SUNITINIB | KIT, RET, CSF1R, ABL1, PDGFRB | PDGFRB, FLT1, KIT, KDR, FLT4, CSF1R | 4.24 |
| PAZOPANIB HYDROCHLORIDE | IMATINIB | FLT1, KDR, FLT4, PDGFRB, KIT | KIT, RET, CSF1R, ABL1, PDGFRB | 4.11 |

**Table S6.** All combinations of drug pairs treated on BT-549 from DrugComb, where one drug targets TUBB1 and the other drug targets PDGFRB.

| Drug 1 | Drug 2 | Drug 1's target(s) | Drug 2's target(s) | ZIP from DrugComb |
| --- | --- | --- | --- | --- |
| PACLITAXEL | SORAFENIB | TUBB1, NR1I2 | FLT4, KDR, PDGFRB, KIT, RET, FLT1 | 18.94 |
| DOCETAXEL | SORAFENIB | TUBB1, NR1I2 | FLT4, KDR, PDGFRB, KIT, RET, FLT1 | 15.81 |
| DOCETAXEL | IMATINIB | TUBB1, NR1I2 | KIT, RET, CSF1R, ABL1, PDGFRB | 14.42 |
| PACLITAXEL | IMATINIB | TUBB1, NR1I2 | KIT, RET, CSF1R, ABL1, PDGFRB | 13.99 |
| PACLITAXEL | PAZOPANIB HYDROCHLORIDE | TUBB1, NR1I2 | FLT1, KDR, FLT4, PDGFRB, KIT | 13.2 |
| DOCETAXEL | SUNITINIB | TUBB1, NR1I2 | PDGFRB, FLT1, KIT, KDR, FLT4, CSF1R | 6.21 |
| CABAZITAXEL | PAZOPANIB HYDROCHLORIDE | TUBB1 | FLT1, KDR, FLT4, PDGFRB, KIT | 4.94 |
| CABAZITAXEL | IMATINIB | TUBB1 | KIT, RET, CSF1R, ABL1, PDGFRB | 2.89 |
| CABAZITAXEL | DASATINIB | TUBB1 | ABL1, KIT, PDGFRB | 2.42 |
| CABAZITAXEL | SUNITINIB | TUBB1 | PDGFRB, FLT1, KIT, KDR, FLT4, CSF1R | 2.18 |
| PACLITAXEL | SUNITINIB | TUBB1, NR1I2 | PDGFRB, FLT1, KIT, KDR, FLT4, CSF1R | −0.19 |
| DOCETAXEL | DASATINIB | TUBB1, NR1I2 | ABL1, KIT, PDGFRB | −0.61 |
| PACLITAXEL | DASATINIB | TUBB1, NR1I2 | ABL1, KIT, PDGFRB | −0.78 |
| CABAZITAXEL | SORAFENIB | TUBB1 | FLT4, KDR, PDGFRB, KIT, RET, FLT1 | −1.17 |

**Table S7.** All combinations of drug pairs treated on BT-549 from DrugComb, where one drug targets TUBB1 and the other drug targets KDR.

| Drug 1 | Drug 2 | Drug 1's target(s) | Drug 2's target(s) | ZIP from DrugComb |
| --- | --- | --- | --- | --- |
| PACLITAXEL | SORAFENIB | TUBB1, NR1I2 | FLT4, KDR, PDGFRB, KIT, RET, FLT1 | 18.94 |
| DOCETAXEL | SORAFENIB | TUBB1, NR1I2 | FLT4, KDR, PDGFRB, KIT, RET, FLT1 | 15.81 |
| PACLITAXEL | AXITINIB | TUBB1, NR1I2 | FLT1, KDR, FLT4 | 14.01 |
| PACLITAXEL | PAZOPANIB HYDROCHLORIDE | TUBB1, NR1I2 | FLT1, KDR, FLT4, PDGFRB, KIT | 13.2 |
| CABAZITAXEL | AXITINIB | TUBB1 | FLT1, KDR, FLT4 | 9.69 |
| DOCETAXEL | AXITINIB | TUBB1, NR1I2 | FLT1, KDR, FLT4 | 6.84 |
| DOCETAXEL | SUNITINIB | TUBB1, NR1I2 | PDGFRB, FLT1, KIT, KDR, FLT4, CSF1R | 6.21 |
| CABAZITAXEL | PAZOPANIB HYDROCHLORIDE | TUBB1 | FLT1, KDR, FLT4, PDGFRB, KIT | 4.94 |
| CABAZITAXEL | SUNITINIB | TUBB1 | PDGFRB, FLT1, KIT, KDR, FLT4, CSF1R | 2.18 |
| CABAZITAXEL | AXITINIB | TUBB1 | FLT1, KDR, FLT4 | −0.15 |
| PACLITAXEL | SUNITINIB | TUBB1, NR1I2 | PDGFRB, FLT1, KIT, KDR, FLT4, CSF1R | −0.19 |
| CABAZITAXEL | SORAFENIB | TUBB1 | FLT4, KDR, PDGFRB, KIT, RET, FLT1 | −1.17 |

**Table S8.** All combinations of drug pairs treated on BT-549 from DrugComb, where one drug targets PDGFRB and the other drug targets KDR.

| Drug 1 | Drug 2 | Drug 1's target(s) | Drug 2's target(s) | ZIP from DrugComb |
| --- | --- | --- | --- | --- |
| DASATINIB | AXITINIB | ABL1, KIT, PDGFRB | FLT1, KDR, FLT4 | 6.04 |
| DASATINIB | PAZOPANIB HYDROCHLORIDE | ABL1, KIT, PDGFRB | FLT1, KDR, FLT4, PDGFRB, KIT | 5.49 |
| IMATINIB | SUNITINIB | KIT, RET, CSF1R, ABL1, PDGFRB | PDGFRB, FLT1, KIT, KDR, FLT4, CSF1R | 4.24 |
| IMATINIB | PAZOPANIB HYDROCHLORIDE | KIT, RET, CSF1R, ABL1, PDGFRB | FLT1, KDR, FLT4, PDGFRB, KIT | 4.11 |
| PAZOPANIB HYDROCHLORIDE | SUNITINIB | FLT1, KDR, FLT4, PDGFRB, KIT | PDGFRB, FLT1, KIT, KDR, FLT4, CSF1R | 3.38 |
| PAZOPANIB HYDROCHLORIDE | AXITINIB | FLT1, KDR, FLT4, PDGFRB, KIT | FLT1, KDR, FLT4 | 3.35 |
| IMATINIB | AXITINIB | KIT, RET, CSF1R, ABL1, PDGFRB | FLT1, KDR, FLT4 | 3.2 |
| SUNITINIB | AXITINIB | PDGFRB, FLT1, KIT, KDR, FLT4, CSF1R | FLT1, KDR, FLT4 | 2.97 |
| PAZOPANIB HYDROCHLORIDE | AXITINIB | FLT1, KDR, FLT4, PDGFRB, KIT | FLT1, KDR, FLT4 | 2.42 |
| DASATINIB | SUNITINIB | ABL1, KIT, PDGFRB | PDGFRB, FLT1, KIT, KDR, FLT4, CSF1R | 0.7 |
| IMATINIB | SORAFENIB | KIT, RET, CSF1R, ABL1, PDGFRB | FLT4, KDR, PDGFRB, KIT, RET, FLT1 | 0.37 |
| PAZOPANIB HYDROCHLORIDE | SORAFENIB | FLT1, KDR, FLT4, PDGFRB, KIT | FLT4, KDR, PDGFRB, KIT, RET, FLT1 | 0.15 |
| DASATINIB | SORAFENIB | ABL1, KIT, PDGFRB | FLT4, KDR, PDGFRB, KIT, RET, FLT1 | −1.71 |
| SORAFENIB | AXITINIB | FLT4, KDR, PDGFRB, KIT, RET, FLT1 | FLT1, KDR, FLT4 | −2.28 |
| SORAFENIB | SUNITINIB | FLT4, KDR, PDGFRB, KIT, RET, FLT1 | PDGFRB, FLT1, KIT, KDR, FLT4, CSF1R | −3.27 |

**Table S9.** All combinations of drug pairs treated on BT-549 from DrugComb, where one drug targets TOP2A and the other drug targets KIT.

| Drug 1 | Drug 2 | Drug 1's target(s) | Drug 2's target(s) | ZIP from DrugComb |
| --- | --- | --- | --- | --- |
| TENIPOSIDE | IMATINIB | TOP2A | KIT, RET, CSF1R, ABL1, PDGFRB | 13.67 |
| DAUNORUBICIN HYDROCHLORIDE | PAZOPANIB HYDROCHLORIDE | TOP2A | FLT1, KDR, FLT4, PDGFRB, KIT | 13.04 |
| MITOXANTRONE | IMATINIB | TOP2A | KIT, RET, CSF1R, ABL1, PDGFRB | 12.47 |
| MITOXANTRONE | PAZOPANIB HYDROCHLORIDE | TOP2A | FLT1, KDR, FLT4, PDGFRB, KIT | 8.56 |
| ADM HYDROCHLORIDE | IMATINIB | TOP2A | KIT, RET, CSF1R, ABL1, PDGFRB | 8.07 |
| MITOXANTRONE | SUNITINIB | TOP2A | PDGFRB, FLT1, KIT, KDR, FLT4, CSF1R | 7.58 |
| MITOXANTRONE | NILOTINIB | TOP2A | ABL1, KIT | 7.4 |
| TENIPOSIDE | NILOTINIB | TOP2A | ABL1, KIT | 5.54 |
| NSC256439 | DASATINIB | TOP2A | ABL1, KIT, PDGFRB | 4.42 |
| ANTIBIOTIC AD 32 | IMATINIB | TOP2A | KIT, RET, CSF1R, ABL1, PDGFRB | 4.38 |
| TENIPOSIDE | DASATINIB | TOP2A | ABL1, KIT, PDGFRB | 3.41 |
| MITOXANTRONE | DASATINIB | TOP2A | ABL1, KIT, PDGFRB | 3.02 |
| ANTIBIOTIC AD 32 | SUNITINIB | TOP2A | PDGFRB, FLT1, KIT, KDR, FLT4, CSF1R | 2.78 |
| ANTIBIOTIC AD 32 | DASATINIB | TOP2A | ABL1, KIT, PDGFRB | 2.3 |
| NSC256439 | SUNITINIB | TOP2A | PDGFRB, FLT1, KIT, KDR, FLT4, CSF1R | 2.08 |
| DEXRAZOXANE | IMATINIB | TOP2A | KIT, RET, CSF1R, ABL1, PDGFRB | 1.49 |
| TENIPOSIDE | SUNITINIB | TOP2A | PDGFRB, FLT1, KIT, KDR, FLT4, CSF1R | 1.36 |
| ANTIBIOTIC AD 32 | NILOTINIB | TOP2A | ABL1, KIT | 1.22 |
| ADM HYDROCHLORIDE | SORAFENIB | TOP2A | FLT4, KDR, PDGFRB, KIT, RET, FLT1 | 0.89 |
| ANTIBIOTIC AD 32 | PAZOPANIB HYDROCHLORIDE | TOP2A | FLT1, KDR, FLT4, PDGFRB, KIT | 0.44 |
| DAUNORUBICIN HYDROCHLORIDE | NILOTINIB | TOP2A | ABL1, KIT | 0.25 |
| MITOXANTRONE | SORAFENIB | TOP2A | FLT4, KDR, PDGFRB, KIT, RET, FLT1 | −0.25 |
| DEXRAZOXANE | NILOTINIB | TOP2A | ABL1, KIT | −0.58 |
| DEXRAZOXANE | SUNITINIB | TOP2A | PDGFRB, FLT1, KIT, KDR, FLT4, CSF1R | −1.67 |
| NSC256439 | NILOTINIB | TOP2A | ABL1, KIT | −1.82 |
| DEXRAZOXANE | PAZOPANIB HYDROCHLORIDE | TOP2A | FLT1, KDR, FLT4, PDGFRB, KIT | −2.22 |
| DAUNORUBICIN HYDROCHLORIDE | DASATINIB | TOP2A | ABL1, KIT, PDGFRB | −3.05 |
| ADM HYDROCHLORIDE | DASATINIB | TOP2A | ABL1, KIT, PDGFRB | −3.21 |
| TENIPOSIDE | SORAFENIB | TOP2A | FLT4, KDR, PDGFRB, KIT, RET, FLT1 | −3.53 |
| DAUNORUBICIN HYDROCHLORIDE | IMATINIB | TOP2A | KIT, RET, CSF1R, ABL1, PDGFRB | −3.7 |
| DAUNORUBICIN HYDROCHLORIDE | SUNITINIB | TOP2A | PDGFRB, FLT1, KIT, KDR, FLT4, CSF1R | −4.5 |
| ADM HYDROCHLORIDE | NILOTINIB | TOP2A | ABL1, KIT | −5.17 |
| ADM HYDROCHLORIDE | SUNITINIB | TOP2A | PDGFRB, FLT1, KIT, KDR, FLT4, CSF1R | −5.5 |
| TENIPOSIDE | PAZOPANIB HYDROCHLORIDE | TOP2A | FLT1, KDR, FLT4, PDGFRB, KIT | −5.89 |
| DEXRAZOXANE | DASATINIB | TOP2A | ABL1, KIT, PDGFRB | −6.2 |
| DEXRAZOXANE | SORAFENIB | TOP2A | FLT4, KDR, PDGFRB, KIT, RET, FLT1 | −7.46 |
| DAUNORUBICIN HYDROCHLORIDE | SORAFENIB | TOP2A | FLT4, KDR, PDGFRB, KIT, RET, FLT1 | −8.5 |

**Table S10.** All combinations of drug pairs treated on BT-549 from DrugComb, where one drug targets TOP2A and the other drug targets CSF1R.

| Drug 1 | Drug 2 | Drug 1's target(s) | Drug 2's target(s) | ZIP from DrugComb |
| --- | --- | --- | --- | --- |
| TENIPOSIDE | IMATINIB | TOP2A | KIT, RET, CSF1R, ABL1, PDGFRB | 13.67 |
| MITOXANTRONE | IMATINIB | TOP2A | KIT, RET, CSF1R, ABL1, PDGFRB | 12.47 |
| ADM HYDROCHLORIDE | IMATINIB | TOP2A | KIT, RET, CSF1R, ABL1, PDGFRB | 8.07 |
| MITOXANTRONE | SUNITINIB | TOP2A | PDGFRB, FLT1, KIT, KDR, FLT4, CSF1R | 7.58 |
| ANTIBIOTIC AD 32 | IMATINIB | TOP2A | KIT, RET, CSF1R, ABL1, PDGFRB | 4.38 |
| ANTIBIOTIC AD 32 | SUNITINIB | TOP2A | PDGFRB, FLT1, KIT, KDR, FLT4, CSF1R | 2.78 |
| NSC256439 | SUNITINIB | TOP2A | PDGFRB, FLT1, KIT, KDR, FLT4, CSF1R | 2.08 |
| DEXRAZOXANE | IMATINIB | TOP2A | KIT, RET, CSF1R, ABL1, PDGFRB | 1.49 |
| TENIPOSIDE | SUNITINIB | TOP2A | PDGFRB, FLT1, KIT, KDR, FLT4, CSF1R | 1.36 |
| DEXRAZOXANE | SUNITINIB | TOP2A | PDGFRB, FLT1, KIT, KDR, FLT4, CSF1R | −1.67 |
| DAUNORUBICIN HYDROCHLORIDE | IMATINIB | TOP2A | KIT, RET, CSF1R, ABL1, PDGFRB | −3.7 |
| DAUNORUBICIN HYDROCHLORIDE | SUNITINIB | TOP2A | PDGFRB, FLT1, KIT, KDR, FLT4, CSF1R | −4.5 |
| ADM HYDROCHLORIDE | SUNITINIB | TOP2A | PDGFRB, FLT1, KIT, KDR, FLT4, CSF1R | −5.5 |

**Table S11.** All combinations of drug pairs treated on BT-549 from DrugComb, where one drug targets KIT and the other drug targets CSF1R.

| Drug 1 | Drug 2 | Drug 1's target(s) | Drug 2's target(s) | ZIP from DrugComb |
| --- | --- | --- | --- | --- |
| NILOTINIB | SUNITINIB | ABL1, KIT | PDGFRB, FLT1, KIT, KDR, FLT4, CSF1R | 4.73 |
| IMATINIB | SUNITINIB | KIT, RET, CSF1R, ABL1, PDGFRB | PDGFRB, FLT1, KIT, KDR, FLT4, CSF1R | 4.24 |
| PAZOPANIB HYDROCHLORIDE | IMATINIB | FLT1, KDR, FLT4, PDGFRB, KIT | KIT, RET, CSF1R, ABL1, PDGFRB | 4.11 |
| DASATINIB | IMATINIB | ABL1, KIT, PDGFRB | KIT, RET, CSF1R, ABL1, PDGFRB | 3.69 |
| PAZOPANIB HYDROCHLORIDE | SUNITINIB | FLT1, KDR, FLT4, PDGFRB, KIT | PDGFRB, FLT1, KIT, KDR, FLT4, CSF1R | 3.38 |
| NILOTINIB | IMATINIB | ABL1, KIT | KIT, RET, CSF1R, ABL1, PDGFRB | 2.79 |
| DASATINIB | SUNITINIB | ABL1, KIT, PDGFRB | PDGFRB, FLT1, KIT, KDR, FLT4, CSF1R | 0.7 |
| SORAFENIB | IMATINIB | FLT4, KDR, PDGFRB, KIT, RET, FLT1 | KIT, RET, CSF1R, ABL1, PDGFRB | 0.37 |
| SORAFENIB | SUNITINIB | FLT4, KDR, PDGFRB, KIT, RET, FLT1 | PDGFRB, FLT1, KIT, KDR, FLT4, CSF1R | −3.27 |

**Table S12.** Examples of drug pairs from which the models potentially captured the synergistic patterns for MCF-7.

| Drug 1 | Drug 2 | Drug 1's target(s) retrieved from DrugBank | Drug 2's target(s) retrieved from DrugBank | ZIP retrieved from DrugComb |
| --- | --- | --- | --- | --- |
| MITOXANTRONE | IMATINIB | TOP2A | KIT, RET, CSF1R, ABL1, PDGFRB | 12.27 |
| MITOXANTRONE | DASATINIB | TOP2A | ABL1, KIT, PDGFRB | 8.38 |
| ANTIBIOTIC AD 32 | IMATINIB | TOP2A | KIT, RET, CSF1R, ABL1, PDGFRB | 6.65 |
| PACLITAXEL | PAZOPANIB HYDROCHLORIDE | TUBB1, NR1I2 | FLT1, KDR, FLT4, PDGFRB, KIT | 8.35 |
| CABAZITAXEL | PAZOPANIB HYDROCHLORIDE | TUBB1 | FLT1, KDR, FLT4, PDGFRB, KIT | 6.48 |
| DOCETAXEL | PAZOPANIB HYDROCHLORIDE | TUBB1, NR1I2 | FLT1, KDR, FLT4, PDGFRB, KIT | 6.16 |
| PACLITAXEL | IMATINIB | TUBB1, NR1I2 | KIT, RET, CSF1R, ABL1, PDGFRB | 6.12 |
| CABAZITAXEL | NILOTINIB | TUBB1 | ABL1, KIT | 3.75 |
| CABAZITAXEL | IMATINIB | TUBB1 | KIT, RET, CSF1R, ABL1, PDGFRB | 3.34 |

**Table S13.** All combinations of drug pairs treated on MCF-7 from DrugComb, where one drug targets TUBB1 and the other drug targets PDGFRB.

| Drug 1 | Drug 2 | Drug 1's target(s) | Drug 2's target(s) | ZIP from DrugComb |
| --- | --- | --- | --- | --- |
| PACLITAXEL | PAZOPANIB HYDROCHLORIDE | TUBB1, NR1I2 | FLT1, KDR, FLT4, PDGFRB, KIT | 8.35 |
| CABAZITAXEL | PAZOPANIB HYDROCHLORIDE | TUBB1 | FLT1, KDR, FLT4, PDGFRB, KIT | 6.48 |
| DOCETAXEL | PAZOPANIB HYDROCHLORIDE | TUBB1, NR1I2 | FLT1, KDR, FLT4, PDGFRB, KIT | 6.16 |
| PACLITAXEL | IMATINIB | TUBB1, NR1I2 | KIT, RET, CSF1R, ABL1, PDGFRB | 6.12 |
| PACLITAXEL | SORAFENIB | TUBB1, NR1I2 | FLT4, KDR, PDGFRB, KIT, RET, FLT1 | 4.47 |
| CABAZITAXEL | IMATINIB | TUBB1 | KIT, RET, CSF1R, ABL1, PDGFRB | 3.34 |
| DOCETAXEL | IMATINIB | TUBB1, NR1I2 | KIT, RET, CSF1R, ABL1, PDGFRB | 1.85 |
| DOCETAXEL | SUNITINIB | TUBB1, NR1I2 | PDGFRB, FLT1, KIT, KDR, FLT4, CSF1R | 1.27 |
| CABAZITAXEL | SUNITINIB | TUBB1 | PDGFRB, FLT1, KIT, KDR, FLT4, CSF1R | −1.15 |
| CABAZITAXEL | SORAFENIB | TUBB1 | FLT4, KDR, PDGFRB, KIT, RET, FLT1 | −1.43 |
| DOCETAXEL | DASATINIB | TUBB1, NR1I2 | ABL1, KIT, PDGFRB | −2.46 |
| DOCETAXEL | SORAFENIB | TUBB1, NR1I2 | FLT4, KDR, PDGFRB, KIT, RET, FLT1 | −2.87 |
| CABAZITAXEL | DASATINIB | TUBB1 | ABL1, KIT, PDGFRB | −2.96 |
| PACLITAXEL | SUNITINIB | TUBB1, NR1I2 | PDGFRB, FLT1, KIT, KDR, FLT4, CSF1R | −3.46 |
| PACLITAXEL | DASATINIB | TUBB1, NR1I2 | ABL1, KIT, PDGFRB | −7.28 |

**Table S14.** All combinations of drug pairs treated on MCF-7 from DrugComb, where one drug targets TUBB1 and the other drug targets ABL1.

| Drug 1 | Drug 2 | Drug 1's target(s) | Drug 2's target(s) | ZIP from DrugComb |
| --- | --- | --- | --- | --- |
| PACLITAXEL | IMATINIB | TUBB1, NR1I2 | KIT, RET, CSF1R, ABL1, PDGFRB | 6.12 |
| CABAZITAXEL | NILOTINIB | TUBB1 | ABL1, KIT | 3.75 |
| CABAZITAXEL | IMATINIB | TUBB1 | KIT, RET, CSF1R, ABL1, PDGFRB | 3.34 |
| DOCETAXEL | NILOTINIB | TUBB1, NR1I2 | ABL1, KIT | 1.9 |
| DOCETAXEL | IMATINIB | TUBB1, NR1I2 | KIT, RET, CSF1R, ABL1, PDGFRB | 1.85 |
| PACLITAXEL | NILOTINIB | TUBB1, NR1I2 | ABL1, KIT | 1.52 |
| DOCETAXEL | DASATINIB | TUBB1, NR1I2 | ABL1, KIT, PDGFRB | −2.46 |
| CABAZITAXEL | DASATINIB | TUBB1 | ABL1, KIT, PDGFRB | −2.96 |
| PACLITAXEL | DASATINIB | TUBB1, NR1I2 | ABL1, KIT, PDGFRB | −7.28 |

**Table S15.** All combinations of drug pairs treated on MCF-7 from DrugComb, where one drug targets TOP2A and the other drug targets PDGFRB.

| Drug 1 | Drug 2 | Drug 1's target(s) | Drug 2's target(s) | ZIP from DrugComb |
| --- | --- | --- | --- | --- |
| MITOXANTRONE | IMATINIB | TOP2A | KIT, RET, CSF1R, ABL1, PDGFRB | 12.27 |
| MITOXANTRONE | DASATINIB | TOP2A | ABL1, KIT, PDGFRB | 8.38 |
| ANTIBIOTIC AD 32 | IMATINIB | TOP2A | KIT, RET, CSF1R, ABL1, PDGFRB | 6.65 |
| TENIPOSIDE | DASATINIB | TOP2A | ABL1, KIT, PDGFRB | 6.4 |
| TENIPOSIDE | IMATINIB | TOP2A | KIT, RET, CSF1R, ABL1, PDGFRB | 6.27 |
| MITOXANTRONE | PAZOPANIB HYDROCHLORIDE | TOP2A | FLT1, KDR, FLT4, PDGFRB, KIT | 5.75 |
| ANTIBIOTIC AD 32 | SUNITINIB | TOP2A | PDGFRB, FLT1, KIT, KDR, FLT4, CSF1R | 5.61 |
| NSC256439 | IMATINIB | TOP2A | KIT, RET, CSF1R, ABL1, PDGFRB | 4.26 |
| NSC256439 | SUNITINIB | TOP2A | PDGFRB, FLT1, KIT, KDR, FLT4, CSF1R | 4.15 |
| ANTIBIOTIC AD 32 | DASATINIB | TOP2A | ABL1, KIT, PDGFRB | 2.78 |
| NSC256439 | PAZOPANIB HYDROCHLORIDE | TOP2A | FLT1, KDR, FLT4, PDGFRB, KIT | 2.59 |
| DAUNORUBICIN HYDROCHLORIDE | PAZOPANIB HYDROCHLORIDE | TOP2A | FLT1, KDR, FLT4, PDGFRB, KIT | 2.55 |
| DEXRAZOXANE | DASATINIB | TOP2A | ABL1, KIT, PDGFRB | 2.53 |
| TENIPOSIDE | SUNITINIB | TOP2A | PDGFRB, FLT1, KIT, KDR, FLT4, CSF1R | 1.92 |
| MITOXANTRONE | SORAFENIB | TOP2A | FLT4, KDR, PDGFRB, KIT, RET, FLT1 | 1.72 |
| ANTIBIOTIC AD 32 | PAZOPANIB HYDROCHLORIDE | TOP2A | FLT1, KDR, FLT4, PDGFRB, KIT | 1.6 |
| MITOXANTRONE | SUNITINIB | TOP2A | PDGFRB, FLT1, KIT, KDR, FLT4, CSF1R | 1.56 |
| NSC256439 | SORAFENIB | TOP2A | FLT4, KDR, PDGFRB, KIT, RET, FLT1 | 1.53 |
| NSC256439 | DASATINIB | TOP2A | ABL1, KIT, PDGFRB | 1.47 |
| DAUNORUBICIN HYDROCHLORIDE | DASATINIB | TOP2A | ABL1, KIT, PDGFRB | 0.92 |
| ANTIBIOTIC AD 32 | SORAFENIB | TOP2A | FLT4, KDR, PDGFRB, KIT, RET, FLT1 | 0.84 |
| TENIPOSIDE | SORAFENIB | TOP2A | FLT4, KDR, PDGFRB, KIT, RET, FLT1 | −0.64 |
| ADM HYDROCHLORIDE | PAZOPANIB HYDROCHLORIDE | TOP2A | FLT1, KDR, FLT4, PDGFRB, KIT | −1.63 |
| ADM HYDROCHLORIDE | SORAFENIB | TOP2A | FLT4, KDR, PDGFRB, KIT, RET, FLT1 | −1.84 |
| TENIPOSIDE | PAZOPANIB HYDROCHLORIDE | TOP2A | FLT1, KDR, FLT4, PDGFRB, KIT | −2.2 |
| ADM HYDROCHLORIDE | IMATINIB | TOP2A | KIT, RET, CSF1R, ABL1, PDGFRB | −2.47 |
| DAUNORUBICIN HYDROCHLORIDE | IMATINIB | TOP2A | KIT, RET, CSF1R, ABL1, PDGFRB | −2.62 |
| DEXRAZOXANE | SUNITINIB | TOP2A | PDGFRB, FLT1, KIT, KDR, FLT4, CSF1R | −4.66 |
| ADM HYDROCHLORIDE | SUNITINIB | TOP2A | PDGFRB, FLT1, KIT, KDR, FLT4, CSF1R | −4.76 |
| DEXRAZOXANE | PAZOPANIB HYDROCHLORIDE | TOP2A | FLT1, KDR, FLT4, PDGFRB, KIT | −5.25 |
| DEXRAZOXANE | IMATINIB | TOP2A | KIT, RET, CSF1R, ABL1, PDGFRB | −5.29 |
| ADM HYDROCHLORIDE | DASATINIB | TOP2A | ABL1, KIT, PDGFRB | −5.74 |
| DAUNORUBICIN HYDROCHLORIDE | SORAFENIB | TOP2A | FLT4, KDR, PDGFRB, KIT, RET, FLT1 | −7.5 |
| DEXRAZOXANE | SORAFENIB | TOP2A | FLT4, KDR, PDGFRB, KIT, RET, FLT1 | −7.84 |
| DAUNORUBICIN HYDROCHLORIDE | SUNITINIB | TOP2A | PDGFRB, FLT1, KIT, KDR, FLT4, CSF1R | −8.07 |

**Table S16.** All combinations of drug pairs treated on MCF-7 from DrugComb, where one drug targets TOP2A and the other drug targets ABL1.

| Drug 1 | Drug 2 | Drug 1's target(s) | Drug 2's target(s) | ZIP from DrugComb |
| --- | --- | --- | --- | --- |
| MITOXANTRONE | IMATINIB | TOP2A | KIT, RET, CSF1R, ABL1, PDGFRB | 12.27 |
| MITOXANTRONE | DASATINIB | TOP2A | ABL1, KIT, PDGFRB | 8.38 |
| ANTIBIOTIC AD 32 | IMATINIB | TOP2A | KIT, RET, CSF1R, ABL1, PDGFRB | 6.65 |
| TENIPOSIDE | DASATINIB | TOP2A | ABL1, KIT, PDGFRB | 6.4 |
| TENIPOSIDE | IMATINIB | TOP2A | KIT, RET, CSF1R, ABL1, PDGFRB | 6.27 |
| NSC256439 | IMATINIB | TOP2A | KIT, RET, CSF1R, ABL1, PDGFRB | 4.26 |
| TENIPOSIDE | NILOTINIB | TOP2A | ABL1, KIT | 3.5 |
| ANTIBIOTIC AD 32 | NILOTINIB | TOP2A | ABL1, KIT | 3.5 |
| NSC256439 | NILOTINIB | TOP2A | ABL1, KIT | 3.4 |
| ANTIBIOTIC AD 32 | DASATINIB | TOP2A | ABL1, KIT, PDGFRB | 2.78 |
| DEXRAZOXANE | DASATINIB | TOP2A | ABL1, KIT, PDGFRB | 2.53 |
| NSC256439 | DASATINIB | TOP2A | ABL1, KIT, PDGFRB | 1.47 |
| DAUNORUBICIN HYDROCHLORIDE | DASATINIB | TOP2A | ABL1, KIT, PDGFRB | 0.92 |
| MITOXANTRONE | NILOTINIB | TOP2A | ABL1, KIT | 0.79 |
| ADM HYDROCHLORIDE | IMATINIB | TOP2A | KIT, RET, CSF1R, ABL1, PDGFRB | −2.47 |
| DAUNORUBICIN HYDROCHLORIDE | IMATINIB | TOP2A | KIT, RET, CSF1R, ABL1, PDGFRB | −2.62 |
| ADM HYDROCHLORIDE | NILOTINIB | TOP2A | ABL1, KIT | −3.3 |
| DAUNORUBICIN HYDROCHLORIDE | NILOTINIB | TOP2A | ABL1, KIT | −4.57 |
| DEXRAZOXANE | IMATINIB | TOP2A | KIT, RET, CSF1R, ABL1, PDGFRB | −5.29 |
| ADM HYDROCHLORIDE | DASATINIB | TOP2A | ABL1, KIT, PDGFRB | −5.74 |
| DEXRAZOXANE | NILOTINIB | TOP2A | ABL1, KIT | −5.87 |

**Table S17.** All combinations of drug pairs treated on MCF-7 from DrugComb, where one drug targets PDGFRB and the other drug targets ABL1.

| Drug 1 | Drug 2 | Drug 1's target(s) | Drug 2's target(s) | ZIP from DrugComb |
| --- | --- | --- | --- | --- |
| PAZOPANIB HYDROCHLORIDE | IMATINIB | FLT1, KDR, FLT4, PDGFRB, KIT | KIT, RET, CSF1R, ABL1, PDGFRB | 3.18 |
| SORAFENIB | IMATINIB | FLT4, KDR, PDGFRB, KIT, RET, FLT1 | KIT, RET, CSF1R, ABL1, PDGFRB | 2.99 |
| SUNITINIB | NILOTINIB | PDGFRB, FLT1, KIT, KDR, FLT4, CSF1R | ABL1, KIT | 2.42 |
| SORAFENIB | NILOTINIB | FLT4, KDR, PDGFRB, KIT, RET, FLT1 | ABL1, KIT | 1.86 |
| PAZOPANIB HYDROCHLORIDE | NILOTINIB | FLT1, KDR, FLT4, PDGFRB, KIT | ABL1, KIT | 1.4 |
| SUNITINIB | IMATINIB | PDGFRB, FLT1, KIT, KDR, FLT4, CSF1R | KIT, RET, CSF1R, ABL1, PDGFRB | 1.26 |
| IMATINIB | NILOTINIB | KIT, RET, CSF1R, ABL1, PDGFRB | ABL1, KIT | 1.12 |
| IMATINIB | DASATINIB | KIT, RET, CSF1R, ABL1, PDGFRB | ABL1, KIT, PDGFRB | 0.58 |
| PAZOPANIB HYDROCHLORIDE | DASATINIB | FLT1, KDR, FLT4, PDGFRB, KIT | ABL1, KIT, PDGFRB | 0.45 |
| DASATINIB | NILOTINIB | ABL1, KIT, PDGFRB | ABL1, KIT | −0.06 |
| SUNITINIB | DASATINIB | PDGFRB, FLT1, KIT, KDR, FLT4, CSF1R | ABL1, KIT, PDGFRB | −0.8 |
| SORAFENIB | DASATINIB | FLT4, KDR, PDGFRB, KIT, RET, FLT1 | ABL1, KIT, PDGFRB | −3.25 |

**Table S18.** Predictive performances of individual leave-out cell lines.

| Leave-out cell line | Correlation coefficient | | | | Mean squared error (MSE) | | | |
| --- | --- | --- | --- | --- | --- | --- | --- | --- |
|  | Protein-inhibition profiles | Protein-inhibition profiles (random weight) | Protein-inhibition profiles (shuffled) | Morgan fingerprint | Protein-inhibition profiles | Protein-inhibition profiles (random weight) | Protein-inhibition profiles (shuffled) | Morgan fingerprint |
| BT-549 | 0.53 | 0.51 | 0.54 | 0.62 | 0.84 | 0.87 | 0.84 | 0.71 |
| MCF-7 | 0.37 | 0.3 | 0.42 | 0.48 | 0.77 | 0.81 | 0.73 | 0.68 |
| MDA-MB-231 | 0.46 | 0.39 | 0.33 | 0.51 | 0.77 | 0.82 | 0.85 | 0.72 |
| MDA-MB-468 | 0.38 | 0.3 | 0.43 | 0.56 | 0.99 | 1.04 | 0.93 | 0.78 |
| T-47D | 0.38 | 0.33 | 0.41 | 0.48 | 0.78 | 0.81 | 0.76 | 0.69 |
| Mean ± SD | 0.42 ± 0.07 | 0.37 ± 0.09 | 0.43 ± 0.08 | 0.53 ± 0.06 | 0.83 ± 0.09 | 0.87 ± 0.10 | 0.82 ± 0.08 | 0.72 ± 0.04 |

**Table S19.** Predictive performances of individual leave-out drugs.

| Leave-out drug | Correlation coefficient | | | | Mean squared error (MSE) | | | |
| --- | --- | --- | --- | --- | --- | --- | --- | --- |
|  | Protein-inhibition profiles | Protein-inhibition profiles (random weight) | Protein-inhibition profiles (shuffled) | Morgan fingerprint | Protein-inhibition profiles | Protein-inhibition profiles (random weight) | Protein-inhibition profiles (shuffled) | Morgan fingerprint |
| ADM HYDROCHLORIDE | 0.27 | 0.29 | 0.24 | 0.27 | 0.65 | 0.64 | 0.68 | 0.65 |
| CRIZOTINIB | 0.55 | 0.55 | 0.41 | 0.62 | 0.61 | 0.61 | 0.85 | 0.6 |
| VEMURAFENIB | 0.39 | 0.41 | 0.32 | 0.61 | 0.94 | 0.95 | 1.19 | 0.87 |
| RALOXIFENE | 0.41 | 0.4 | 0.41 | 0.39 | 0.61 | 0.58 | 0.6 | 0.59 |
| ANASTROZOLE | 0.32 | 0.43 | 0.37 | 0.53 | 0.5 | 0.41 | 0.43 | 0.36 |
| AXITINIB | 0.54 | 0.38 | 0.57 | 0.53 | 0.67 | 0.77 | 0.68 | 0.66 |
| VANDETANIB | 0.54 | 0.33 | 0.36 | 0.54 | 0.72 | 0.84 | 0.83 | 0.71 |
| VISMODEGIB | 0.39 | 0.38 | 0.29 | 0.56 | 1.08 | 0.95 | 1.18 | 0.86 |
| VINBLASTINE SULFATE | 0.45 | 0.5 | 0.47 | 0.49 | 0.6 | 0.58 | 0.63 | 0.56 |
| BORTEZOMIB | 0.34 | 0.45 | 0.33 | 0.37 | 1.09 | 1.02 | 1.18 | 1.1 |
| EMCYT (PHARMACIA) | 0.4 | 0.41 | 0.33 | 0.36 | 1.64 | 1.74 | 1.8 | 1.88 |
| NSC256439 | 0.4 | 0.33 | 0.31 | 0.35 | 1.09 | 1.15 | 1.23 | 1.15 |
| ACTINOMYCIN D | 0.41 | 0.44 | 0.4 | 0.4 | 2.49 | 2.51 | 2.81 | 2.61 |
| DOCETAXEL | 0.48 | 0.55 | 0.52 | 0.68 | 1.68 | 1.68 | 1.76 | 1.27 |
| PACLITAXEL | 0.58 | 0.58 | 0.6 | 0.74 | 1.44 | 1.45 | 1.56 | 1.15 |
| 1-(5-DEOXYPENTOFURANOSYL)-5-FLUORO-4-{[(PENTYLOXY)CARBONYL]AMINO}PYRIMIDIN-2(1H)-ONE | 0.4 | 0.48 | 0.29 | 0.49 | 0.49 | 0.5 | 0.6 | 0.48 |
| 34793-34-5 | 0.24 | 0.3 | 0.18 | 0.35 | 0.79 | 0.66 | 0.7 | 0.68 |
| 5-FU | 0.4 | 0.4 | 0.4 | 0.54 | 1.23 | 1.23 | 1.09 | 1.06 |
| IMATINIB | 0.46 | 0.47 | 0.41 | 0.58 | 0.6 | 0.6 | 0.73 | 0.63 |
| SN-38 | 0.42 | 0.44 | 0.29 | 0.43 | 1.1 | 1.1 | 1.13 | 1.03 |
| TENIPOSIDE | 0.39 | 0.41 | 0.24 | 0.49 | 1.27 | 1.29 | 1.55 | 1.25 |
| MITOXANTRONE | 0.46 | 0.51 | 0.44 | 0.5 | 1.69 | 1.52 | 1.81 | 1.52 |
| PAZOPANIB HYDROCHLORIDE | 0.51 | 0.48 | 0.33 | 0.48 | 0.96 | 0.84 | 1.16 | 0.9 |
| SUNITINIB | 0.38 | 0.42 | 0.32 | 0.51 | 0.68 | 0.78 | 0.71 | 0.68 |
| DASATINIB | 0.34 | 0.35 | 0.29 | 0.31 | 0.79 | 0.78 | 0.84 | 0.82 |
| Mean ± SD | 0.42 ± 0.08 | 0.43 ± 0.08 | 0.36 ± 0.10 | 0.48 ± 0.12 | 1.02 ± 0.48 | 1.01 ± 0.49 | 1.11 ± 0.54 | 0.96 ± 0.49 |
